# Impact of offensive team variables on goal scoring in the first division of the spanish soccer league: a comprehensive 10-year study

**DOI:** 10.1038/s41598-024-77199-8

**Published:** 2024-10-24

**Authors:** Pablo Prieto-González, Víctor Martín, Martin Pacholek, Alejandro Sal-de-Rellán, Rui Marcelino

**Affiliations:** 1 Sport Sciences and Diagnostics Research Group, GSD-HPE Department, Prince Sultan University, Riyadh, Saudi Arabia; 2 Faculty of Health Sciences, Universidad Isabel I de Castilla, Burgos, Spain; 3 Faculty of Social Sciences and Communication, Universidad Europea de Madrid, Madrid, Spain; 4 Research Center in Sports Sciences, Creative Lab Research Community, Health Sciences and Human Development, CIDESD, Vila Real, Portugal; 5 University of Maia, Maia, Portugal; 6 Portugal Football School, Portuguese Football Federation, Oeiras, Portugal

**Keywords:** Football, Offensive metrics, Goal-scoring metrics, Team performance, Match analysis, Physiology, Health care

## Abstract

This study assessed the impact of team offensive variables on goals scored in Spain’s first-division soccer league from 2012–2013 to 2021–2022. A nomothetic multidimensional study was conducted, following the STROBE checklist. From 115 team variables available in the INSTAT database, 57 offensive metrics were selected. The selection was performed independently by five researchers to ensure comprehensive inclusion of relevant metrics. The data included 38 league matches each season, involving 30 teams. The study identified key offensive performance metrics strongly correlated with goals scored. These include shots on target (r = 0.898), chances created (r = 0.871), penalty box entries (r = 0.852), positional attacks with shots (r = 0.818), total shots (r = 0.807), central attacks with shots (r = 0.804), accurate passes (r = 0.760), and efficiency in positional attacks (r = 0.755). The findings underscore the importance of specific offensive metrics in enhancing goal-scoring capabilities. Coaches can improve their team’s offensive performance by focusing on passing accuracy, dribbling, and maximizing scoring chances. Successful teams consistently convert attacks into shots on goal through various situations, including central attacks, flanks, counterattacks, corners, and set pieces. Tactical adjustments based on these insights can optimize offensive effectiveness.

## Introduction

Soccer is a high-intensity intermittent team sport^1^ in which many variables can influence success. Previous studies have shown that players’ technical level, physical condition, team tactics, and mentality are key factors in team performance^2^. Moreover, these factors are all influenced by contextual variables^3^. Likewise, the analysis of soccer performance has shown that the offensive phase is a central element of its dynamics. In this phase, a team’s success is determined by its ability to score goals^4,5^. Consequently, these are dependent on and influenced by team´s contribution to game actions^6^. Understanding the key factors driving game outcomes is essential for identifying potential barriers to scoring goals in matches and understanding the intricate interplay of elements influencing soccer matches^7^.

With advancements in technology, soccer researchers have access to a vast amount of data on the performance of soccer players. This information helps identify the key variables to attain team’s success. Match and video analysis provide valuable qualitative and quantitative insights into individual player performance and overall team dynamics^8^. Additionally, utilizing data provided by InStat, a renowned and reliable sports analytics platform known for its comprehensive coverage of soccer matches, provides a useful dataset for understanding the relationship between offensive metrics and goal outcomes^9^.

As a result, video analysts play a crucial role in gathering and analyzing game metrics to assess specific playing styles and patterns and explore different game contexts and strategies adopted by opponents^10^. Several factors, like the quality of the opposing team, the match’s outcome, and the game’s location, are related to success in a match^3,11^. More specifically, factors such as ball possession, accurate passes, number of successful passes, number of shots on goal, and shooting efficiency are vital aspects of the game that can determine success in a match^12,13^. Teams with superior offensive attributes often demonstrate higher success rates in metrics such as shots on goal and goal conversion^4,14^, underscoring the significance of offensive prowess over defensive strategies.

Numerous studies have examined scoring opportunities to identify factors influencing match outcomes in European soccer leagues^15,16^. Additionally, Sarmento et al.^17^ analyzed playing styles in the English Premier League, La Liga, and Italian Serie A, revealing distinct characteristics of each league, with La Liga demonstrating a dominant aesthetic style characterized by superior game control^17^. Although many of these studies have focused on analyzing the relationships between offensive parameters and goal scoring, particularly in the English League, a notable gap exists in investigating these aspects in the Spanish League. Despite being considered the best league in international prestige and due to the competitive quality of its teams between 2012 and 2016^18^, it remains underrepresented in research compared to the Premier League^19^. Addressing this gap through scientific research is essential^20,21^.

Furthermore, previous studies have frequently analyzed goal-scoring patterns using a restricted set of variables, and the criteria for including these variables are not specified. However, currently, with the significant volume of data and variables available through platforms such as InStat, it is possible to investigate the factors influencing a team’s goal-scoring capability and offensive performance success indicators. Understanding the dynamics of team offensive variables and their correlation with goals scored is crucial for elucidating the success of soccer teams. However, achieving this necessitates a comprehensive analysis to gain a deeper understanding and offer valuable insights for coaches and analysts. Therefore, this study aimed to determine the influence of team offensive variables on goals scored in the first division of the Spanish soccer league between 2012–2013 and 2021–2022. Through this analysis, the authors aimed to enhance comprehension of the determinants of offensive success in soccer. Based on similar articles^6,12,13^, it was hypothesized that accurate passes, ball possession, number of successful passes, number of shots on goal, and shooting efficiency are the key variables that most significantly contribute to soccer success.

## Methodology

### Study design

A nomothetic multidimensional study was conducted, with adherence to the ethical principles outlined in the Declaration of Helsinki. Approval was obtained from the Institutional Review Board at Prince Sultan University, Saudi Arabia (PSU IRB-2024-05-0182), ensuring the study’s integrity. The study was conducted in accordance with the STROBE (Strengthening the Reporting of Observational Studies in Epidemiology) checklist.

### Setting & participants &variables & data sources/ measurement

From a pool of 115 team variables available in the INSTAT database (accessed on September 15, 2022), encompassing both offensive and defensive metrics, 57 variables were selected for inclusion in the study (see Table 1 and Supplementary Material 1).

**Table 1:** Descriptive Statistics of Variables included in the study: Average per season over the 10 seasons (2012–2013 to 2021–2022) in the First Division of La Liga (38 Match days per Season).

Variable	X̅ ± SD	IC 95% Lower	IC 95% Upper	Variable	X̅ ± SD	IC 95% Lower	IC 95% Upper
Accurate passes	14,739.83 ± 3321.972	14,184.72	15,294.93	Offsides	83.22 ± 17.00	80.38	86.06
Attacking challenges won	1405.87 ± 194.21	1373.42	1438.33	Passes	17,949.23 ± 3206.11	17,413.48	18,484.97
Attacks – center	802.97 ± 120.59	782.82	823.12	Penalties	6.24 ± 2.71	5.78	6.69
Attacks—left flank	1038.17 ± 119.52	1018.20	1058.15	Percentage of accurate crosses	25.60 ± 2.92	25.12	26.09
Attacks—right flank	1068.22 ± 118.99	1048.33	1088.10	Percentage of accurate passes	81.53 ± 3.93	80.87	82.19
Attacks with shots—center	97.77 ± 29.934	92.77	102.78	Percentage of challenges in attack won	46.27 ± 2.82	45.79	46.74
Attacks with shots—left flank	92.88 ± 22.79	89.07	96.69	Percentage of efficiency for attacks through the central zone	12.11 ± 2.68	11.65	12.54
Attacks with shots—right flank	91.27 ± 20.85	87.79	94.76	Percentage of efficiency for attacks through the left flank	8.94 ± 1.89	8.62	9.25
Attacks with shots—Set pieces attacks	111.25 ± 17.98	108.24	114.25	Percentage of efficiency for attacks through the right flank	8.55 ± 1.82	8.23	8.86
Average duration of ball possession (sec)	14.81 ± 2.51	14.37	15.22	Percentage of efficiency for corner attacks	29.67 ± 4.33	28.94	30.39
Ball possession percentage	49.84 ± 5.65	48.89	50.78	Percentage of efficiency for counterattacks	14.52 ± 3.20	13.99	15.06
Ball possessions quantity	4006.23 ± 212.69	3970.69	4041.77	Percentage of efficiency for free-kick attacks	34.33 ± 7.99	32.99	35.67
Chances	187.03 ± 51.19	178.34	195.23	Percentage of efficiency for positional attacks	8.75 ± 1.91	8.43	9.07
Chances percentage	26.61 ± 4.36	25.87	27.32	Percentage of efficiency for set-piece attacks	31.47 ± 3.78	30.84	32.11
Corner attacks	179.82 ± 29.45	174.90	184.74	Percentage of efficiency for throw-in attacks	16.30 ± 10.11	14.61	17.99
Corners	179.81 ± 29.70	174.83	184.76	Percentage of penalties scored	75.90 ± 19.19	72.70	79.11
Counter-attacks	527.40 ± 80.29	513.98	540.82	Percentage of shots on target	39.17 ± 3.47	38.59	39.75
Counter-attacks with a shot	76.01 ± 17.28	73.12	78.90	Percentage of successful dribbles	57.44 ± 3.84	56.80	58.08
Crosses	502.24 ± 104.77	484.73	519.75	Percentage scored free kick shots	5.351 ± 4.61	4.57	6.12
Crosses accurate	129.37 ± 34.17	123.66	135.08	Positional attacks	2381.91 ± 171.93	2353.24	2410.70
Dribbles	1016.35 ± 197.84	983.29	1049.40	Positional attacks with shots	209.28 ± 50.88	200.78	217.78
Entrances to the final third	1495.15 ± 187.25	1463.86	1526.44	Set pieces attacks	354.92 ± 50.74	346.44	363.40
Entrances to the opposition half	2343.03 ± 213.09	2307.42	2378.64	Shots	416.79 ± 73.23	404.55	429.03
Entrance to the penalty box	552.82 ± 119.30	532.88	572.75	Shots on post / bar	9.94 ± 3.96	9.23	10.56
Free-kick attacks	138.82 ± 34.02	133.13	144.50	Shots on target	163.98 ± 38.29	157.58	170.38
Free-kick shots	20.35 ± 9.39	18.78	21.92	Shots wide	152.81 ± 24.54	148.71	156.91
Goals	50.05 ± 17.09	47.19	52.90	Successful dribbles	587.84 ± 143.88	563.79	611.88
Lost balls	2701.01 ± 186.51	2669.84	2732.18	Throw-in attacks	28.92 ± 23.73	24.96	32.89
Lost balls in own half	537.32 ± 70.35	525.56	549.07				

${\overline{\text{X}}}$: mean; SD: standard deviation; min: minimum; max: maximum; IC 95% Lower: Lower bound of the 95% Confidence Interval; IC 95% Upper: Upper bound of the 95% Confidence Interval.

Only offensive variables were considered due to their demonstrated significance as indicators of offensive team success, as established in previous research^4^. The five main researchers conducted the selection process independently and unthinkingly and identified exclusively offensive variables from the available pool. Only those offensive variables selected by at least four of the five main researchers, representing a threshold of 80%, were included in the study.

The data utilized in this study pertained to the first division of the Spanish Soccer League.

The dataset spans a period of 10 years from the seasons 2012–2013 to 2021–2022, covering 38 league matches each season. Given that the Spanish soccer league comprises 20 teams in its first division, the total number of matches analyzed was 3800. Additionally, considering that the bottom three teams in the league are relegated at the end of each season, and three new teams from the second division join the first division, the teams vary slightly each season. Over the 10 years, the total number of teams analyzed amounted to 30.

### Bias

The intra-class correlation coefficient (ICC) was used to assess the consistency of all team offensive parameters throughout the 10 seasons analyzed. Consistently high ICC values were observed across all parameters, with single measures equal to or exceeding 0.896 and average measures equal to or surpassing 0.984 for each parameter. These outcomes suggest robust reliability in the league data spanning from the 2012–2013 to the 2021–2022 seasons.

### Statistical analyses

Data are presented as mean ± standard deviation (SD). The normality of the data and the residuals were assessed using the Kolmogorov–Smirnov test, and the homoscedasticity of variances was examined utilizing the Levene test. Pearson’s bivariate correlation coefficient was employed to analyze the correlation between variables. The results were interpreted as follows: 0 ≤ r ≤ 0.09 very weak; 0.10 ≤ r ≤ 0.29 weak; 0.30 ≤ r ≤ 0.49 moderate; 0.50 ≤ r ≤ 0.69; strong; and r ≥ 0.70 very strong^22^. A regression analysis was conducted to determine the nature of the association between goals scored (dependent variable) and the selected team offensive variables (predictor variables). Subsequently, scatter plots were examined to assess whether a linear or non-linear regression model was appropriate. It was found that multiple linear regression provided the best fit, indicating a robust association between the variables under investigation. Following this, the independence of residuals was evaluated using the Durbin-Watson statistic, with values falling within the range of 1.5 to 2.5 considered acceptable for indicating independence^23^. Afterward, multicollinearity between the predictor variables was assessed using the Variance Inflation Factor, with a threshold of 10 sets, and collinearity tolerance, with a threshold of at least 0.1, to identify substantial correlations between variables^24^. Furthermore, the Bayesian Information Criterion and the Akaike Information Criterion were utilized to address the potential problem of overfitting due to the strong correlation found among specific variables (see Supplementary Material 2). These criteria penalize excessive model complexity, facilitating the selection of a more parsimonious model capable of effectively generalizing to unseen data while avoiding including redundant or insignificant variables, thus enhancing the model’s generalization ability. Given the large number of predictor variables included, the stepwise method was chosen for regression analysis^25^. Correlation analysis was considered to assess relationships between predictor variables and dependent variables to identify the best-fit models. Models were then constructed with various predictor combinations, evaluating each model’s performance using the R-squared, Mean Squared Error, and Mean Absolute Error metrics. Variables showing low correlation and insignificance in coefficient tests were considered for removal, ensuring model efficiency and eliminating redundancy^26^. Data analysis was performed using IBM SPSS Statistics (Version 26) software.

## Results

Once the data’s normality and homoscedasticity were confirmed, the Durbin-Watson statistical values fell between 1.5 and 2.5. Similarly, the Variance Inflation Factor values were below 10, and collinearity tolerance values were higher than 0.1. This confirmed that the fundamental assumptions for multiple regression analysis were met. As for Pearson’s bivariate correlation results (see Table 2), the following variables demonstrated a very strong positive and significant correlation with goals scored in this sequence: shots on target, chances, entrance to the penalty box, positional attacks with shots, shots, attacks with shots—center, accurate passes, passes, and percentage of efficiency for positional attacks.

**Table 2:** Correlation between the team offensive variables selected and the goals scored.

Variable	*r*	*p*	Variable	*r*	*p*
Accurate passes	0.760*	< 0.001	Offsides	0.578*	< 0.001
Attacking challenges won	− 0.011	0.897	Passes	0.759*	< 0.001
Attacks – center	0.502*	< 0.001	Penalties	0.363*	< 0.001
Attacks—left flank	0.065	0.446	Percentage of accurate crosses	0.048	0.574
Attacks—right flank	-0.098	0.249	Percentage of accurate passes	0.647*	< 0.001
Attacks with shots—center	0.804*	< 0.001	Percentage of challenges in attack won	0.540*	< 0.001
Attacks with shots—left flank	0.592*	< 0.001	Percentage of efficiency for attacks through the central zone	0.698*	< 0.001
Attacks with shots—right flank	0.594*	< 0.001	Percentage of efficiency for attacks through the left flank	0.620*	< 0.001
Attacks with shots—Set pieces attacks	0.307*	< 0.001	Percentage of efficiency for attacks through the right flank	0.688*	< 0.001
Average duration of ball possession (sec)	0.696*	< 0.001	Percentage of efficiency for corner attacks	− 0.076	0.37
Ball possession percentage	0.697*	< 0.001	Percentage of efficiency for counterattacks	0.672*	< 0.001
Chances	0.871*	< 0.001	Percentage of efficiency for free-kick attacks	0.672*	< 0.001
Chances percentage	0.511*	< 0.001	Percentage of efficiency for positional attacks	0.755*	< 0.001
Corner attacks	0.585*	< 0.001	Percentage of efficiency for set-piece attacks	0.537*	< 0.001
Corners	0.591*	< 0.001	Percentage of efficiency for throw-in attacks	− 0.137	0.107
Counter-attacks	− 0.165	0.051	Percentage of penalties scored	0.089	0.295
Counter-attacks with a shot	0.521*	< 0.001	Percentage of shots on target	0.636*	< 0.001
Crosses	− 0.15	0.078	Percentage of successful dribbles	0.500*	< 0.001
Crosses accurate	− 0.09	0.293	Percentage scored free kick shots	0.151	0.077
Dribbles	0.534*	< 0.001	Positional attacks	0.406*	< 0.001
Entrance to the penalty box	0.852*	< 0.001	Positional attacks with shots	0.818*	< 0.001
Entrances to the final third	0.670*	< 0.001	Set pieces attacks	− 0.104	0.221
Entrances to the opposition half	0.431*	< 0.001	Shots	0.807*	< 0.001
Free-kick attacks	− 0.411*	< 0.001	Shots on post / bar	0.636*	< 0.001
Free-kick shots	0.505*	< 0.001	Shots on target	0.898*	< 0.001
Lost balls	− 0.492*	< 0.001	Shots wide	0.487*	< 0.001
Lost balls in own half	− 0.113	0.182	Successful dribbles	0.585*	< 0.001
Offsides	0.578*	< 0.001	Throw-in attacks	− 0.416*	< 0.001
Passes	0.759*	< 0.001			

*r:* Coefficient of Correlation; *p*: Significance Level.

*Significant correlation found; n = 200.

These variables also displayed a significant strong positive correlation in the following order: Percentage of efficiency for attacks through the central zone, ball possession percentage, the average duration of ball possession (sec), percentage of efficiency for attacks through the right flank, percentage of efficiency for counterattacks, percentage of efficiency for free-kick attacks, entrances to the final third, percentage of accurate passes, percentage of shots on target, shots on post/bar, percentage of efficiency for attacks through the left flank, attacks with shots—right flank, attacks with shots—left flank, corners, corner attacks, successful dribbles, offsides, percentage of challenges in attack won, percentage of efficiency for set-piece attacks, dribbles, counterattacks with a shot, chances percentage, Free-kick shots, Attacks—center, Percentage of successful dribbles.

The following variables exhibit a significant positive moderate correlation with goals in the order in which they are listed: shots wide, entrances to the opposition half, throw-in attacks, free-kick attacks, positional attacks, penalties, attacks with shots—set-piece attacks. These variables showed a significant and moderate negative correlation in this sequence: lost balls, throw-in attacks, free-kick attacks. Finally, variables including percentage scored free kick shots, percentage of penalties scored, attacks—left flank, percentage of accurate crosses, successful tackles, attacking challenges won, percentage of efficiency for corner attacks, crosses accurate, attacks—right flank, set pieces attacks, lost balls in own half, percentage of efficiency for throw-in attacks, crosses, ball possessions quantity, and counterattacks showed no correlation with goals scored.

**Fig. 1:**
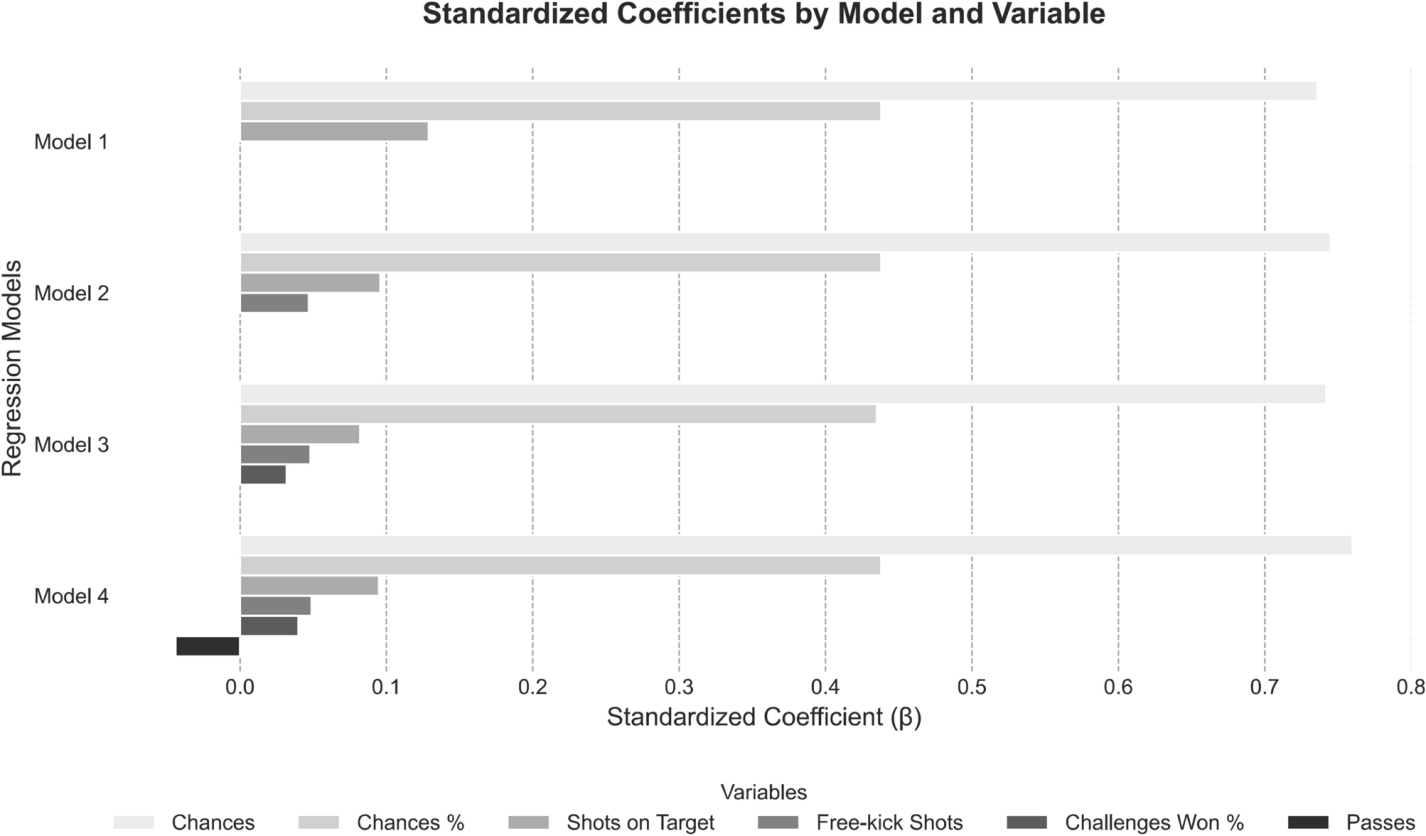
Stepwise multilinear regression analysis of the association between goals scored and team offensive variables.

Furthermore, four models were generated in the multiple regression analysis, with goals scored serving as the dependent variable and the remaining team offensive variables as the independent variables (see Fig. 1). Model 1 demonstrated a high coefficient of determination (R^2^ = 0.981) with significant predictors, including chances (β = 0.736, p < 0.001), chances percentage (β = 0.438, p < 0.001), and shots on target (β = 0.129, p < 0.001), listed in decreasing order of importance. Model 2 improved slightly, achieving R^2^ = 0.982, and included chances (β = 0.745, p < 0.001), chances percentage (β = 0.438, p < 0.001), shots on target (β = 0.096, p = 0.001), and free-kick shots (β = 0.047, p < 0.001). Model 3 showed a further increase in explanatory power (R^2^ = 0.983) and included chances (β = 0.742, p < 0.001), chances percentage (β = 0.435, p < 0.001), shots on target (β = 0.082, p = 0.005), free-kick shots (β = 0.048, p < 0.001), and percentage of challenges in attack won (β = 0.032, p = 0.016). Finally, Model 4 achieved the highest explanatory power (R^2^ = 0.984), incorporating chances (β = 0.760, p < 0.001), chances percentage (β = 0.438, p < 0.001), shots on target (β = 0.095, p = 0.001), free-kick shots (β = 0.049, p < 0.001), passes (β = − 0.044, p = 0.024), and percentage of challenges in attack won (β = 0.040, p = 0.004), ranked in decreasing order of importance.

## Discussion

The present research aimed to determine the influence of team offensive variables on goals scored in the first division of the Spanish soccer league between the 2012–2013 and 2021–2022 seasons. Shots on target, chances, entrance to the penalty box, positional attacks with shots, shots, attacks with shots/center, accurate passes, passes, and percentage of efficiency for positional attacks were identified as key offensive metrics. These findings underscore the importance of both the quality and quantity of passing, emphasizing a focus on positional and tactical play, as well as the ability to create scoring opportunities by penetrating through the center and into the penalty area. The team’s success ultimately depends on finishing these chances with precision, highlighting the technical and tactical skills necessary for success in soccer.

In the literature on offensive performance in soccer, goal scoring is considered the ultimate determinant of offensive effectiveness^15^. The results of this study confirm that shots**,** particularly shots on target**,** are crucial for scoring goals and influencing match outcomes, aligning with previous findings^46^. Studies show that top teams tend to generate more shots and shots on goal than lower-ranked teams^14,16^, and the shot-to-goal ratio is a critical factor distinguishing successful teams^13,27^. However, the effectiveness of shots depends on factors such as location and context^28^. Additionally, the present research highlights the importance of chances and entrance to the penalty box, reinforcing that a multidimensional analysis of goal attempts—considering variables like shooting distance, angle, and defensive pressure—provides a deeper understanding of offensive success^15^.

Furthermore, passes and accurate passes, along with the ability to convert plays into shots on goal—particularly through positional play and central attacks**—**had a significant impact on goal-scoring success. Passing accuracy is a key factor in offensive performance, as supported by several studie^6,29–33^. However, in the English Premier League, a more direct style of play is associated with greater offensive success compared to other leagues^29,30^. Regarding attacking flanks, the study´s findings align with those of Wang & Qin (2020)^34^, who observed that a high percentage of shots and goals are initiated from the central flank.

Based on the results, shots on target, chances percentage, and chances were consistently identified as key variables across most models, emphasizing their crucial role in offensive performance. This suggests that a team’s success largely depends on its ability to create and capitalize on scoring opportunities. Previous studies have shown that winning teams typically record higher numbers of shots, chances, and shot efficiency than less successful teams^14,35^. Additionally, research highlights that in around 70% of matches, the team scoring the first goal usually wins^4,36^, underlining the importance of offensive strength over defensive factors^14^.

Other variables closely linked to goals scored include attacks with shots from the left and right flanks, corner attacks, counterattacks, free-kick shots, and various percentages of efficiency for attacks in central and flank zones. This emphasizes that a team’s ability to convert plays into shots on goal and the accuracy of those shots are vital for offensive success. Previous studies^4,14^, indicate that teams with lower offensive success tend to exhibit reduced accuracy in their shots on goal compared to more successful teams.

Moreover, entrances to the final third, corners, offsides, and central attacks are important factors related to goals scored, emphasizing the significance of frequently penetrating the opponent’s goal area. These findings somewhat align with those of Guimarães et al. (2022)^37^, who noted a positive association between central corridor entries and successful shots on goal, while side entries showed no such link. Additionally, the present study confirms that more successful teams generate more corner kick opportunities^6,38^. Lago-Ballesteros and Lago-Peñas (2010)^14^ found no significant differences in corner counts among teams in different league positions, nor did they observe variations in offsides, contrary to the present findings.

The present research indicated that possession-related metrics—such as ball possession percentage, average duration of possession, and percentage of accurate passes—are important factors influencing goal scoring and contribute to overall offensive success. This association can distinguish top teams from mid- and lower-table teams, highlighting the technical proficiency of successful teams^14,33^. While ball possession is a widely studied metric^39^, there is no consensus on its relationship with scoring performance. The results partially contrast with previous findings that, although passing accuracy relates to goal success, it does not correlate with the total number of passes^29,30^. This discrepancy may reflect differences in positional play across European leagues. Additionally, teams with lower offensive success tend to experience more lost balls, indicating a reduced technical level. These findings align with those of Ruan et al. (2023) who found that teams with less offensive success made more defensive errors^40^. In contrast, Brito Souza et al. (2019)^6^ found no significant link between lost balls and points scored in the Spanish league. Interestingly, Dellal et al. (2011)^41^ observed that professional teams have fewer ball losses per possession compared to amateur teams and demonstrate improved passing precision.

The importance of attempting shots at the goal is underscored by the positive relationship between shots wide and goals scored. This suggests that teams employing an offensive strategy, consistently targeting the opposition’s goal—even if some shots miss—tend to be more successful. These findings contrast with those of Castellano et al. (2012)^13^, who found that victorious teams did not take more shots wide compared to those that drew or lost. However, it is important to recognize that shots wide, often viewed negatively in soccer analysis and assigned a value of 0 in player evaluations, account for a significant portion—approximately two-thirds—of total shots^42^. Thus, shots wide can provide valuable insights into players’ shooting capabilities, allowing for more accurate assessments^42^.

Moreover, entries into the opposition half are linked to greater goal-scoring success, emphasizing the importance of maintaining possession and progressing toward the opposing goal to create more scoring opportunities. These findings are consistent with previous research^43^, which indicated that entries into the opposition’s half decrease when playing away or against stronger opponents, while they tend to increase against weaker teams. In contrast to the negative impact of throw-in attacks, the positive impact of positional attacks on goals scored suggests that teams employing structured positional play can generate more scoring opportunities. This indicates that teams with greater offensive success effectively execute plays from stable positions, while those with lower performance often resort to a more direct style. In this context, Stone and Smith (2021)^44^ noted that during the 2018–2019 English Premier League season, only 54% of throw-ins resulted in retained possession, and just 8.8% led to a shot on goal after a successful first contact.

Both free-kick attacks and penalties are linked to goals scored, likely because highly successful attacking teams generate more scoring opportunities, prompting opponents to commit fouls. While the frequency of free-kick attacks and penalties awarded in soccer has received limited research attention, it has been observed that teams finishing higher in the league standings and home teams tend to receive more penalties^6,45^, Additionally, local teams often score more goals than visiting teams^12^. Thus, the findings regarding penalties awarded align with the limited existing literature on the topic, although further research is needed to confirm this aspect.

Furthermore, the association between attacks with shots from set pieces and goals scored highlights that teams achieving the highest offensive success can generate numerous strategic actions and effectively convert them into shots on target. Goals scored from set pieces constitute a significant portion of overall goals in various competitions^34,46^. However, the relationship between this variable and offensive scoring success has not been the focus of recent research in the Spanish league. Notably, Miraballes(2017)^47^, found no significant association between the performance of semifinalist teams in the 2017 South Korea U-20 Soccer World Cup and their ability to convert set pieces into shots on goal. This indicates a need for future studies to address this research gap.

While the percentage of goals from free kicks and penalties can be significant in individual matches, they represent only 10.68% and 12.46% of total goals scored across a season, rendering them less impactful overall (see Table 1). Similarly, the efficiency in set-piece attacks may be relevant in individual matches, but it does not significantly influence the total number of goals scored. This finding aligns with those reported by Guimarães et al. (2021)^37^. The absence of a link between attacks from the left and right flanks and goals scored indicates that central attacks are more decisive, likely due to the wider angle to the goal; however, they require higher technical proficiency. This finding aligns with previous research^37^ and may clarify why over half of the attacks in the Spanish league originate from the center^48^.

Crosses, counterattacks, and their accuracy, which showed no significant link to goals scored, suggest that teams may adopt a more direct style due to lower passing quality and possession. This pattern aligns with previous studies in the Spanish and Greek leagues^14,38^, indicating that top teams do not necessarily rely on crosses. Furthermore, research suggests that higher crossing accuracy may result in fewer crosses to avoid offside traps^49^. However, Sarmento et al. (2018) indicated that the offensive efficiency of counterattacks was 40% higher than that of positional attacks in the Spanish, Italian, and English Leagues^50^.

The absence of an association between attacking challenges won and goals scored suggests that this metric may reflect physical condition and aggressiveness rather than technical quality. Teams that win more challenges may have a higher number of contested balls and less ball control, which is often seen in those with lower technical skills. Additionally, the number of lost balls in their half showed no connection to scoring, likely because successful teams take more passing risks to create opportunities. Further research is needed to validate these observations.

This study has several limitations: (1) it focuses solely on the first division of the Spanish soccer league, which may limit its applicability to other leagues; (2) external factors such as match conditions, injuries, and opposing defensive patterns were not considered, despite their potential influence on team performance; (3) individual factors like age, experience, and fatigue were excluded, as the primary objective was to explore team dynamics; and (4) qualitative data from players or coaches were not incorporated, which could have provided valuable context to the findings. Furthermore, the analysis spans a decade, and given the rapid evolution of soccer tactics, this historical context may affect relevance. Future studies could benefit from dividing the data into shorter time frames, such as two 5-year periods, to better capture tactical evolutions and enhance the understanding of goal-scoring phenomena and team performance dynamics. Additionally, future research should consider integrating individual player characteristics and defensive patterns of opposing teams. Contextual factors, such as pitch areas and attack lengths, should also be addressed, as they may influence the collective attacking strategies analyzed in this study.

## Conclusion

This study highlights the essential role of specific offensive metrics in achieving goal-scoring success in the Spanish league from 2012–2013 to 2021–2022. The findings indicate that teams emphasizing effective passing, accurate shooting, and the ability to create scoring opportunities by penetrating through the center and into the penalty area are more likely to succeed. Quality and quantity in passing and dribbling are crucial for generating these opportunities, significantly enhancing a team’s scoring chances. Additionally, accuracy in finishing plays is vital, as successful teams have a greater ability to convert various scoring situations, including attacks from different areas, counterattacks, corner kicks, and set pieces. Therefore, understanding the dynamics of offensive success in the Spanish league relies on the technical proficiency of the teams. Mastering these fundamental elements enables teams to improve their performance and achieve greater success.

### Practical applications

The findings of this study offer valuable insights for soccer coaches, analysts, and teams in enhancing offensive performance. Coaches can use key offensive metrics such as shots on target and chances created to develop strategic training plans, focusing on these aspects to improve overall team effectiveness. Emphasizing passing accuracy and precision in finishing within player development programs will provide players with the necessary skills and decision-making abilities for optimal offensive play.

Teams can adjust tactics based on correlations between offensive variables and goals scored, prioritizing center attacks and possession in attacking zones for increased scoring opportunities. Analysts can assess opponents’ offensive strengths and weaknesses to develop effective defensive strategies. Coaches can integrate these insights into match preparation, devising game plans that capitalize on advantages and minimize threats. Key offensive metrics can also inform player recruitment, targeting individuals with strengths in passing accuracy, chance creation, and finishing to bolster offensive capabilities.

Finally, coaches can utilize the most influential offensive metrics to evaluate players’ progress throughout matches and over the season. Monitoring players’ performance in these areas can identify areas for improvement and track development over time, ultimately contributing to team success.

## Supplementary Information

Supplementary Information 1.

Supplementary Information 2.

## Data Availability

The data supporting the findings of this study, "Impact of Offensive Team Variables on Goal Scoring in the First Division of the Spanish Soccer League: A Comprehensive 10-Year Study," are sourced from the INSTAT database. Due to licensing restrictions, these data are not publicly available. However, they can be obtained from the authors upon reasonable request and with the permission of INSTAT. Access to the data is subject to approval from the relevant authorities. For inquiries regarding data access, please contact the corresponding author, Pablo Prieto-González (pprieto@psu.edu.sa).
