# Supplementary material for: Impact of offensive team variables on goal scoring in the first division of the spanish soccer league: a comprehensive 10-year study
Source: Sci Rep. 2024 Oct 24;14:25231. doi: 10.1038/s41598-024-77199-8 (PMC11502709; doi:10.1038/s41598-024-77199-8)
Supplement: Supplementary file 1 — Supplementary Information 1. [file 41598_2024_77199_MOESM1_ESM.pdf]

Supplementary material 1: Team offensive variables included in the study, along with their definition

Accurate passes	Number of passes successfully completed to a teammate
Attacking challenges won	Number of successful challenges or duels in attacking situations
Attacks - center	Number of offensive actions initiated through the central area of the field
Attacks - left flank	Number of offensive actions initiated through the left side of the field
Attacks - right flank	Number of offensive actions initiated through the right side of the field
Attacks with shots - center	Number of attacking actions resulting in shots initiated through the central area of the field
Attacks with shots - left flank	Number of attacking actions resulting in shots initiated through the left side of the field
Attacks with shots - right flank	Number of attacking actions resulting in shots initiated through the right side of the field
Attacks with shots - Set pieces attacks	Number of attacking actions resulting in shots initiated from set-piece situations
Average duration of ball possession (sec)	Average time the team holds possession of the ball in seconds
Ball possession percentage	Percentage of time the team has possession of the ball
Chances	Total number of scoring opportunities created
Chances percentage	Percentage of successful scoring opportunities created
Corner attacks	Number of attacking actions initiated from corner kick situations
Corners	Number of corner kicks taken
Counter-attacks	Open play attack after the opponent team loses the ball. A counter-attack lasts no longer than 30 sec. Speed of moving to the target in counter-attack - not less than 2,6 meters/sec
Counter-attacks with a shot	Number of counter-attacks resulting in a shot on goal
Crosses	Number of crosses attempted
Crosses accurate	Number of crosses that reach their intended target
Dribbles	Active action of a player possessing the ball, an attempt to pass opponent by using dribbling. When dribbling succeeds, the opponent has unsuccessful tackle registered
Entrance to the penalty box	Number of times the team enters the opponent's penalty area
Entrances to the final third	Number of times the team enters the final third of the pitch
Entrances to the opposition half	Number of times the team enters the opponent's half of the pitch
Free-kick attacks	Number of attacking actions initiated from free-kick situations
Free-kick shots	Number of shots taken from free-kick situations
Goals	Number of goals scored
Lost ball	Player's actions (i.e., inaccurate pass, lost challenge) that lead to losing a ball. Loss of the ball is not registered if the ball possession is finished with a foul suffered or a shot
Lost balls in own half:	Number of possessions lost by the team in their own half of the pitch
Offsides	Number of offside infractions committed by the team

Passes	Total number of passes attempted by the team
Penalties	Number of penalty kicks taken by the team
Percentage of accurate crosses	Percentage of crosses that reach their intended target
Percentage of accurate passes	Percentage of passes successfully completed to a teammate
Percentage of challenges in attack won	Percentage of successful challenges or duels in attacking situations
Percentage of efficiency for attacks through the central zone	Percentage of successful attacking actions initiated through the central area of the field
Percentage of efficiency for attacks through the left flank	Percentage of successful attacking actions initiated through the left side of the field
Percentage of efficiency for attacks through the right flank	Percentage of successful attacking actions initiated through the right side of the field
Percentage of efficiency for corner attacks	Percentage of successful attacking actions initiated from corner kick situations
Percentage of efficiency for counterattacks	Percentage of successful counter-attacks
Percentage of efficiency for free-kick attacks	Percentage of successful attacking actions initiated from free-kick situations
Percentage of efficiency for positional attacks	Percentage of successful attacking actions initiated from a structured position on the field
Percentage of efficiency for set-piece attacks	Percentage of successful attacking actions initiated from set-piece situations
Percentage of efficiency for throw-in attacks	Percentage of successful attacking actions initiated from throw-in situations
Percentage of penalties scored	Percentage of penalty kicks converted into goals
Percentage of shots on target	Percentage of shots that are on target
Percentage of successful dribbles	Percentage of successful attempts to bypass opponents with the ball under control
Percentage scored free kick shots	Percentage of shots taken from free-kicks that result in goals
Positional attacks	Attacks in open play or after set pieces (throw-in or free-kick). Positional attack in open play either lasts more than 30 sec or speed of moving to the target in ball possession is less than 2,6 meters/sec
Positional attacks with shots	Number of attacking actions resulting in shots initiated from a structured position on the field
Set pieces attacks	Number of attacking actions initiated from set-piece situations
Shot	Sending ball to the opponent's goal with the purpose to score
Shots on post / bar	Number of shots that hit the goalpost or crossbar
Shots on target	Number of shots that are on target
Shots wide	Number of shots that miss the target
Successful dribbles	Number of successful attempts to bypass opponents with the ball under control
Throw-in attacks	Number of attacking actions initiated from throw-in situations
