# Supplementary material for: Impact of offensive team variables on goal scoring in the first division of the spanish soccer league: a comprehensive 10-year study
Source: Sci Rep. 2024 Oct 24;14:25231. doi: 10.1038/s41598-024-77199-8 (PMC11502709; doi:10.1038/s41598-024-77199-8)
Supplement: Supplementary file 2 — Supplementary Information 2. [file 41598_2024_77199_MOESM2_ESM.pdf]

Table 3. Correlation matrix among all team offensive game variables.

	Goals	Chances	Chances percentage	Offsides	Corners	Shots	Shots on target	Passes	Percentage of accurate passes	Crosses	Percentage of accurate crosses	Percentage of challenges in attack won	Dribbles	Percentage of successful dribbles	Lost balls	Lost balls in own half	Ball possession percentage	Entrances to the opposition half	Entrances to the final third	Entrance to the penalty box	Percentage of shots on target	Shots wide	Shots on post / bar	Accurate passes	Crosses accurate	Positional attacks	Positional attacks with shots	Percentage of efficiency for positional attacks	Counter-attacks	Counter-attacks with a shot	Percentage of efficiency for counterattacks	Set pieces attacks	Attacks with shots - Set pieces attacks	Percentage of efficiency for set-piece attacks	Free-kick attacks	Percentage of efficiency for free-kick attacks	Corner attacks	Percentage of efficiency for corner attacks	Throw-in attacks	Percentage of efficiency for throw-in attacks	Free-kick shots	Percentage scored free kick shots	Penalties	Percentage of penalties scored	Average duration of ball possession (sec)	Attacks - left flank	Attacks with shots - left flank	Percentage of efficiency for attacks through the left flank	Attacks - center	Attacks with shots - center	Percentage of efficiency for attacks through the central zone	Attacks - right flank	Attacks with shots - right flank	Percentage of efficiency for attacks through the right flank	Attacking challenges won
Chances	.871**																																																						
Chances percentage	.511**	0.05																																																					
Offsides	.578**	.524**	.253**																																																				
Corners	.591**	.608**	0.142	.550**																																																			
Shots	.807**	.795**	.228**	.582**	.759**																																																		
Shots on target	.898**	.878**	.279**	.601**	.681**	.927**																																																	
Passes	.759**	.776**	.217**	.447**	.543**	.719**	.789**																																																
Percentage of accurate passes	.647**	.695**	0.136	.275**	.370**	.592**	.656**	.872**																																															
Crosses	-0.15	-0.101	-0.138	0	.383**	0.146	-0.036	-0.159	-.251**																																														
Percentage of accurate crosses	0.048	0.107	-0.13	-0.066	0.024	0.141	0.085	0.069	0.044	.220**																																													
Percentage of challenges in attack won	.540**	.476**	.222**	.420**	.388**	.493**	.540**	.552**	.320**	-0.092	0.043																																												
Dribbles	.534**	.349**	.431**	.459**	.405**	.524**	.558**	.540**	.409**	-.199*	-0.042	.608**																																											
Percentage of successful dribbles	.500**	.437**	.235**	.284**	.248**	.384**	.454**	.585**	.533**	-.277**	-0.069	.652**	.558**																																										
Lost balls	-.492**	-.661**	0.124	-0.096	-.177*	-.386**	-.464**	-.526**	-.726**	.322**	0.006	-.225**	-0.046	-.394**																																									
Lost balls in own half	-0.113	-.230**	.193*	-0.148	-.293**	-.235**	-0.162	0.101	.304**	-.305**	-0.163	-0.076	.260**	.169*	-0.015																																								
Ball possession percentage	.697**	.723**	.180*	.418**	.559**	.683**	.729**	.935**	.824**	-0.075	0.124	.463**	.477**	.531**	-.467**	0.073																																							
Entrances to the opposition half	.431**	.338**	.252**	.500**	.684**	.580**	.511**	.386**	0.005	.392**	0.158	.457**	.436**	0.146	.351**	-.443**	.414**																																						
Entrances to the final third	.670**	.654**	.206*	.589**	.868**	.824**	.760**	.625**	.347**	.390**	0.137	.498**	.487**	.270**	-0.066	-.411**	.639**	.866**																																					
Entrance to the penalty box	.852**	.883**	.178*	.629**	.788**	.898**	.910**	.790**	.630**	0.079	0.161	.575**	.530**	.429**	-.426**	-.269**	.756**	.616**	.865**																																				
Percentage of shots on target	.636**	.609**	.260**	.344**	.225**	.342**	.662**	.563**	.493**	-.349**	-0.079	.345**	.342**	.356**	-.379**	0.091	.490**	0.132	.277**	.499**																																			
Shots wide	.487**	.478**	0.104	.417**	.660**	.827**	.617**	.464**	.350**	.459**	.260**	.357**	.360**	.202*	-0.145	-.238**	.449**	.559**	.719**	.658**	-0.083																																		
Shots on post / bar	.636**	.634**	.173*	.486**	.459**	.603**	.637**	.539**	.384**	-0.079	0.035	.428**	.432**	.316**	-.244**	-.231**	.487**	.433**	.546**	.643**	.378**	.381**																																	
Accurate passes	.760**	.788**	.201*	.424**	.516**	.712**	.787**	.995**	.911**	-.190*	0.068	.519**	.521**	.584**	-.591**	0.126	.930**	.314**	.584**	.779**	.569**	.448**	.524**																																
Crosses accurate	-0.09	-0.025	-0.161	-0.029	.318**	.183*	0.012	-0.094	-.178*	.899**	.616**	-0.05	-.181*	-.256**	.254**	-.328**	-0.005	.385**	.378**	0.143	-.313**	.485**	-0.038	-0.118																															
Positional attacks	.406**	.297**	.291**	.461**	.576**	.522**	.480**	.515**	.206*	.291**	.189*	.319**	.483**	.214*	.319**	-.183*	.579**	.872**	.766**	.554**	.180*	.485**	.407**	.456**	.315**																														
Positional attacks with shots	.818**	.835**	.197*	.528**	.695**	.940**	.912**	.828**	.741**	0.056	0.148	.467**	.502**	.449**	-.514**	-0.098	.781**	.455**	.749**	.888**	.429**	.745**	.580**	.831**	0.111	.484**																													
Percentage of efficiency for positional attacks	.755**	.810**	0.114	.433**	.591**	.876**	.841**	.758**	.754**	0.006	0.108	.404**	.398**	.428**	-.649**	-0.059	.695**	.246**	.599**	.797**	.382**	.690**	.501**	.777**	0.05	.252**	.957**																												
Counter-attacks	-0.165	-.356**	.223**	0.13	0.081	-0.025	-0.12	-.389**	-.605**	.293**	-0.067	0.044	.198*	-.171*	.757**	-.213*	-.405**	.474**	.176*	-0.108	-.251**	0.12	0.005	-.447**	.206*	.264**	-.246**	-.351**																											
Counter-attacks with a shot	.521**	.381**	.377**	.496**	.428**	.637**	.565**	.317**	.218**	0.035	-0.126	.363**	.446**	.347**	-0.088	-0.148	.242**	.385**	.481**	.495**	0.152	.526**	.366**	.304**	-0.025	.276**	.469**	.435**	.403**																										
Percentage of efficiency for counterattacks	.672**	.670**	.238**	.415**	.390**	.692**	.693**	.632**	.649**	-.172*	-0.061	.368**	.329**	.499**	-.620**	-0.038	.556**	0.099	.402**	.611**	.362**	.461**	.394**	.654**	-0.16	0.128	.675**	.707**	-.274**	.756**																									
Set pieces attacks	-0.104	-0.069	-0.146	0.109	.335**	0.116	-0.008	-.267**	-.460**	.437**	.186*	0.082	-0.075	-.306**	.359**	-.552**	-.185*	.520**	.369**	0.122	-.272**	.247**	-0.012	-.307**	.431**	.215*	-0.078	-0.149	.384**	-0.051	-.313**																								
Attacks with shots - Set pieces attacks	.307**	.333**	-0.009	.263**	.552**	.566**	.449**	0.146	0.006	.385**	.238**	.257**	.174*	-0.077	0.023	-.459**	.207*	.566**	.591**	.470**	-0.016	.579**	.289**	0.128	.419**	.383**	.359**	.270**	.187*	0.16	0.039	.691**																							
Percentage of efficiency for set-piece attacks	.537**	.537**	0.155	.230**	.345**	.632**	.626**	.522**	.555**	-0.007	0.123	.260**	.323**	.261**	-.398**	0.039	.498**	0.15	.366**	.502**	.318**	.494**	.383**	.545**	0.057	.258**	.587**	.553**	-.206*	.284**	.441**	-.244**	.525**																						
Free-kick attacks	-.411**	-.418**	-0.153	-.167*	-.217*	-.309**	-.370**	-.551**	-.654**	.193*	0.149	-0.095	-.209*	-.405**	.497**	-.358**	-.456**	.183*	-0.097	-.336**	-.345**	-0.159	-.243**	-.577**	.218**	-0.022	-.470**	-.509**	.369**	-.261**	-.522**	.754**	.377**	-.388**																					
Percentage of efficiency for free-kick attacks	.672**	.630**	.237**	.361**	.422**	.673**	.722**	.670**	.637**	-.193*	0.092	.359**	.481**	.397**	-.479**	0.04	.635**	.219**	.445**	.616**	.453**	.434**	.480**	.688**	-0.104	.337**	.676**	.620**	-.248**	.319**	.512**	-.224**	.397**	.809**	-.436**																				
Corner attacks	.585**	.602**	0.144	.548**	.999**	.756**	.676**	.541**	.367**	.383**	0.015	.382**	.403**	.244**	-.172*	-.290**	.556**	.683**	.864**	.780**	.218**	.657**	.456**	.514**	.314**	.579**	.691**	.587**	0.086	.434**	.394**	.324**	.544**	.345**	-.225**	.418**																			
Percentage of efficiency for corner attacks	-0.076	-0.015	-0.095	-0.114	0.017	0.104	-0.01	-0.125	-0.073	.310**	0.118	0.038	-0.157	-0.145	0.044	-0.081	-0.137	-0.003	0.012	-0.01	-.220**	.260**	-0.029	-0.121	.304**	-0.076	-0.022	-0.007	0.066	0.022	-0.037	0.119	.436**	.437**	0.079	-0.083	0.019																		
Throw-in attacks	-.416**	-.343**	-.296**	-.238**	-.246**	-.297**	-.385**	-.480**	-.527**	.183*	0.149	-.168*	-.392**	-.396**	.258**	-.295**	-.467**	-0.044	-.194*	-.285**	-.406**	-0.094	-.273**	-.495**	.215*	-.271**	-.392**	-.347**	.167*	-.290**	-.414**	.618**	.198*	-.436**	.451**	-.405**	-.253**	0.097																	
Percentage of efficiency for throw-in attacks	-0.137	-0.126	-0.114	-0.148	-.202*	-0.085	-0.099	-0.116	-0.083	0.007	0.06	-.208*	-0.091	-.172*	0.129	0.108	-0.104	-0.086	-0.137	-.183*	-0.085	-0.061	-0.091	-0.114	0.024	-0.015	-0.076	-0.058	0.013	-0.05	-0.077	-0.075	-0.101	-0.05	0.021	-0.071	-.200*	-0.144	0.07																
Free-kick shots	.505**	.431**	.194*	.282**	.280**	.485**	.538**	.429**	.358**	-.282**	0.078	.289**	.447**	.326**	-.266**	-0.095	.464**	.286**	.355**	.422**	.319**	.250**	.401**	.438**	-.190*	.326**	.459**	.393**	-0.086	0.154	.226**	0.083	.434**	.480**	0.023	.713**	.273**	-.250**	-.235**	0.004															
Percentage scored free kick shots	0.15	0.111	0.109	-0.002	.206*	0.13	.170*	.173*	0.117	-0.004	-0.012	0.108	0.119	0.022	-0.002	-0.008	0.154	.191*	.197*	0.163	.169*	-0.001	0.129	0.162	-0.004	.185*	0.136	0.085	-0.012	-0.03	-0.017	0.07	0.128	0.086	-0.053	.234**	.207*	-.170*	-0.042	-0.014	.252**														
Penalties	.363**	.460**	-0.096	0.153	.232**	.327**	.398**	.246**	.281**	-0.035	0.117	0.078	-0.006	0.07	-.313**	-.242**	.302**	0.114	.260**	.396**	.336**	0.161	.194*	.265**	0.028	0.09	.336**	.325**	-.231**	-0.007	.171*	0.007	.305**	.396**	-0.15	.287**	.218**	0.103	-0.155	-0.104	.221**	0.037													
Percentage of penalties scored	0.089	0.075	0.084	-0.057	-0.001	0.059	0.049	0.103	0.123	-0.014	-0.047	0.049	-0.077	-0.008	-0.121	0.097	.169*	-0.038	0.013	0.065	0.021	0.072	0.071	0.11	-0.024	0.024	0.104	0.095	-.166*	-0.061	0.066	-0.106	0.004	0.13	-0.083	0.095	0.006	0.097	-0.093	-0.042	0.053	0.024	0.055												
Average duration of ball possession (sec)	.696**	.778**	0.098	.293**	.402**	.614**	.706**	.926**	.927**	-.258**	0.057	.418**	.380**	.565**	-.736**	0.128	.886**	0.107	.436**	.691**	.557**	.329**	.437**	.951**	-.179*	.256**	.766**	.765**	-.601**	.203*	.654**	-.385**	0.035	.511**	-.603**	.641**	.398**	-0.124	-.480**	-0.122	.406**	0.134	.299**	0.134											
Attacks - left flank	0.065	0.023	0.045	.227**	.389**	.326**	.188*	0.088	-0.135	.502**	0.12	0.133	.185*	0.042	.393**	-.223**	0.131	.603**	.531**	.276**	-.173*	.468**	0.096	0.04	.452**	.588**	.244**	0.108	.403**	.220**	-0.036	.350**	.364**	0.075	0.093	0.054	.390**	0.111	0.103	-0.034	0.021	0.034	-0.01	-0.011	-0.114										
Attacks with shots - left flank	.592**	.609**	0.132	.397**	.587**	.801**	.719**	.539**	.479**	.258**	0.124	.327**	.359**	.328**	-.342**	-0.139	.502**	.367**	.632**	.689**	.219**	.710**	.362**	.542**	.260**	.357**	.804**	.795**	-0.056	.535**	.609**	-0.049	.318**	.501**	-.399**	.477**	.584**	0.121	-.290**	-0.047	.230**	0.069	.276**	0.057	.485**	.496**									
Percentage of efficiency for attacks through the left flank	.620**	.662**	0.121	.322**	.447**	.734**	.702**	.560**	.606**	0.046	0.119	.282**	.285**	.337**	-.562**	-0.044	.503**	0.113	.435**	.619**	.316**	.573**	.347**	.586**	0.085	0.119	.779**	.841**	-.264**	.495**	.706**	-.242**	.170*	.533**	-.488**	.507**	.443**	0.094	-.391**	-0.018	.242**	0.032	.307**	0.087	.599**	0.053	.876**								
Attacks - center	.502**	.364**	.339**	.401**	.216*	.383**	.480**	.471**	.303**	-.454**	-0.012	.381**	.584**	.343**	0.003	-0.015	.406**	.433**	.337**	.428**	.419**	0.146	.475**	.448**	-.367**	.482**	.374**	.251**	.168*	.322**	.229**	-0.065	0.104	.221**	-0.086	.449**	.218**	-.284**	-.315**	-0.045	.573**	.181*	0.054	-0.012	.346**	-0.098	0.066	0.135							
Attacks with shots - center	.804**	.730**	.321**	.543**	.498**	.792**	.827**	.735**	.632**	-.338**	-0.032	.500**	.605**	.512**	-.441**	-0.037	.643**	.387**	.569**	.754**	.484**	.503**	.567**	.735**	-.283**	.394**	.801**	.745**	-0.112	.590**	.701**	-0.126	.216*	.453**	-.371**	.654**	.495**	-.191*	-.385**	-0.084	.576**	0.109	.190*	0.034	.664**	0.016	.489**	.542**	.706**						
Percentage of efficiency for attacks through the central zone	.698**	.697**	.196*	.445**	.511**	.800**	.763**	.661**	.648**	-0.127	-0.031	.392**	.418**	.444**	-.561**	0.001	.575**	.216*	.521**	.706**	.329**	.594**	.406**	.675**	-0.114	.202*	.825**	.837**	-.243**	.595**	.798**	-0.16	.179*	.453**	-.470**	.543**	.509**	-0.047	-.321**	-0.087	.304**	0.009	.182*	0.064	.642**	0.109	.641**	.660**	.239**	.841**					
Attacks - right flank	-0.098	-.203*	.182*	0.119	.277**	0.022	-0.063	-0.083	-.282**	.575**	0.119	-0.029	0.054	-.195*	.575**	-.169*	0.02	.535**	.351**	0.016	-0.16	0.163	0.015	-0.138	.511**	.544**	-0.09	-.236**	.481**	0.123	-.196*	.284**	.208*	-0.065	.211*	-.191*	.281**	0.111	-0.063	0.067	-.187*	0.042	-0.071	-0.054	-.273**	.217*	-0.087	-.197*	-0.105	-.238**	-.225**				
Attacks with shots - right flank	.594**	.577**	.220**	.471**	.665**	.780**	.681**	.605**	.514**	.370**	0.165	.373**	.357**	.281**	-.257**	-0.135	.597**	.482**	.698**	.704**	.199*	.745**	.496**	.594**	.374**	.464**	.761**	.712**	0.007	.543**	.567**	0.014	.339**	.440**	-.362**	.429**	.668**	0.099	-.305**	-0.033	.168*	0.076	.191*	0.079	.503**	.219**	.575**	.531**	0.107	.460**	.572**	.346**			
Percentage of efficiency for attacks through the right flank	.688**	.724**	0.14	.434**	.559**	.821**	.764**	.697**	.682**	0.071	0.123	.428**	.370**	.421**	-.562**	-0.087	.638**	.262**	.577**	.747**	.302**	.693**	.526**	.711**	0.117	.236**	.856**	.875**	-.228**	.520**	.706**	-0.13	.255**	.503**	-.488**	.562**	.559**	0.022	-.291**	-0.056	.296**	0.079	.248**	0.072	.685**	0.131	.663**	.667**	.187*	.622**	.727**	-0.134	.869**		
Attacking challenges won	-0.011	-.209*	.275**	.199*	0.102	0.037	0.006	-0.067	-.361**	0.089	0.136	.525**	.527**	.195*	.600**	-0.083	-0.07	.589**	.271**	0.062	-0.082	0.123	0.102	-0.134	0.131	.485**	-0.109	-.271**	.650**	.199*	-.234**	.413**	.249**	-0.147	.414**	-0.096	0.101	0.03	0.153	-0.078	0.084	0.035	-.223**	-0.114	-.319**	.366**	-0.099	-.302**	.380**	0.056	-.184*	.387**	-0.012	-.187*	
Successful dribbles	.585**	.414**	.417**	.459**	.399**	.542**	.590**	.604**	.475**	-.241**	-0.036	.694**	.972**	.729**	-0.145	.240**	.538**	.410**	.481**	.562**	.381**	.359**	.449**	.587**	-.213*	.461**	.538**	.444**	0.119	.459**	.402**	-0.13	0.141	.344**	-.270**	.514**	.395**	-0.164	-.416**	-0.129	.471**	0.103	0.03	-0.066	.465**	.171*	.386**	.324**	.580**	.644**	.465**	-0.014	.365**	.416**	.500**

**: p-value is ≤ 0.01.*: p-value is ≤ 0.05
